# Electrophysiology of Memory-Updating Differs with Age

**DOI:** 10.3389/fnagi.2016.00136

**Published:** 2016-06-16

**Authors:** Genevieve Z. Steiner, Craig J. Gonsalvez, Frances M. De Blasio, Robert J. Barry

**Affiliations:** ^1^The National Institute of Complementary Medicine (NICM), Western Sydney UniversityPenrith, NSW, Australia; ^2^Centre for Psychophysics, Psychophysiology, and Psychopharmacology, Brain & Behaviour Research Institute, and School of Psychology, University of WollongongWollongong, NSW, Australia; ^3^School of Social Sciences and Psychology, Western Sydney UniversityPenrith, NSW, Australia

**Keywords:** P3(00), aging, working memory, event-related potentials (ERPs), older adults, healthy aging, target-to-target interval (TTI), oddball task

## Abstract

In oddball tasks, the P3 component of the event-related potential systematically varies with the time between target stimuli—the target-to-target interval (TTI). Longer TTIs result in larger P3 amplitudes and shorter latencies, and this pattern of results has been linked with working memory-updating processes. Given that working memory and the P3 have both been shown to diminish with age, the current study aimed to determine whether the linear relationship between P3 and TTI is compromised in healthy aging by comparing TTI effects on P3 amplitudes and latencies, and reaction time (RT), in young and older adults. Older adults were found to have an overall reduction in P3 amplitudes, longer latencies, an anterior shift in topography, a trend toward slower RTs, and a flatter linear relationship between P3 and TTI than young adults. Results suggest that the ability to maintain templates in working memory required for stimulus categorization decreases with age, and that as a result, neural compensatory mechanisms are employed.

## Introduction

### Electrophysiology of memory-updating differs with age

Working memory, the ability to hold and manipulate information in the mind, decreases across the lifespan (Hedden and Gabrieli, 2004), particularly after 70 years of age (Hester et al., 2004). This age-related cognitive decline can result in a loss of confidence, independence, and reduced quality of life. The decline in working memory ability is thought to result from a reduction in regional brain volume and cortical thickness due to a loss of synaptic density, particularly in the prefrontal cortex (Kadota et al., 2001; Pieperhoff et al., 2008; Salat et al., 2009; Bishop et al., 2010). In order to devise strategies to mitigate the pathophysiology of the aging process, an increased understanding of age-related changes in brain function is required.

The P3(00) component of the human event-related potential (ERP), a large positive deflection elicited by salient stimuli, has been described as an electrophysiological index of working memory processes (Squires et al., 1976, 1977; Polich, 1997). Previous work has shown that by extending the time between target stimuli (the target-to-target interval [TTI]), the P3 increases in amplitude and decreases in latency (Gonsalvez et al., 1999, 2007; Gonsalvez and Polich, 2002; Croft et al., 2003; Steiner et al., 2013a,b; Steiner et al., 2014a,b). This “TTI effect” on the P3 has been directly linked with working memory processes (Gonsalvez et al., 2007), where young adults with better working memory ability showed a steeper increase in P3 amplitude with TTI than those with poorer working memory (Steiner et al., 2013a).

The template-update model has been proposed as a framework to account for the TTI effect on the P3 (Gonsalvez et al., 2007). Here, a stimulus activates a profile, or template, of neural activity in working memory that degrades over time. The template will be updated when the stimulus is next presented (longer TTIs = greater update). In the template-update model, a greater increase in P3 amplitude with longer TTIs indicates more effective activation of working memory processes.

To date, the TTI effect has been explored only in young adults. To address this, the present study examined the TTI effect in older adults, and whether the linear relationship between P3 and TTI would vary with age. It was hypothesized that a flatter linear relationship (P3 as a function of TTI) would be observed for older adults, interpreted by the template-update model as indicating less effective electrophysiological memory-updating processes.

## Methods

### Participants

Participants were 19 students (young adults; mean age = 21.2, *SD* = 3.7, range = 18–35 years, 14 females, 18 right-handed) from the University of Wollongong and 22 older adults (mean age = 68.1, *SD* = 4.2, range = 59–74 years, 16 females, 21 right-handed) from an independent living aged-care facility. Young adults received course credit for participating, and older adults were reimbursed $40 (Australian) for their time. Prior to commencing the experiment, participants provided informed consent, and were free to withdraw at any time without penalty. Individuals self-reporting neurological or psychiatric illnesses, and/or use of psychotropic medication, were excluded. It should be noted that although care was taken to exclude participants taking psychoactive medications (e.g., selective serotonin reuptake inhibitors), most older adults took a range of medications for various other health conditions, for example perindopril, celecoxib, rani 2, nonsteroidal anti-inflammatories, lomotil, diabex, esomeprazole, glucosamine etc. Older participants were also screened for cognitive impairment with the Rowland Universal Dementia Assessment Scale (RUDAS; Conforti et al., 2006); all scored > 22 point cut-off (*M* = 28.0, *SD* = 1.5). Self-reports indicated that participants had refrained from psychoactive substances for at least 12 h and from tea, coffee, alcohol, and cigarettes for at least 2 h prior to testing. All participants had normal or corrected-to-normal vision and self-reported normal hearing.

### Procedure

A demographic and screening questionnaire was completed by all participants before they were fitted with electroencephalogram (EEG) recording apparatus. Prior to the experiment, participants completed an electrooculogram (EOG)/EEG calibration task (Croft and Barry, 2000). Participants were seated 600–800 mm in front of a 48.3 cm (19″) Dell LCD monitor and instructed to fixate on a 10 × 10 mm black cross centered on a gray background.

The experimental task was a visual oddball paradigm (target probability = 30%, nontarget probability = 70%) broken into four different blocks (approximately 3 min each), with short rest intervals between blocks to minimize fatigue. Stimuli consisted of 45 × 45 mm black “tick” (target) and “cross” (nontarget) images, each presented for 300 ms on a gray background with a fixed 1.5 s ISI. The TTI was manipulated 1.5–12.0 s, with 30 trials presented for each TTI of interest (1.5, 3.0, 6.0, and 9.0 s); a total of 532 stimuli were presented in a randomized sequence. To balance possible speed/accuracy trade-offs, participants were instructed to “respond to target stimuli with a button press, as quickly and as accurately as possible.” Participants responded with their dominant hand on a Logitech® Precision game controller. Instruction was given to sit as still as possible, but participants were not directly instructed to refrain from blinking (Verleger, 1991). This procedure was approved by the joint South Eastern Sydney/Illawarra Area Health Service and University of Wollongong Health and Medical Human Research Ethics Committee.

### Materials and apparatus

EEG data were recorded continuously DC–70 Hz from A2 and 19 scalp sites (Fp1, Fp2, F7, F3, Fz, F4, F8, T3, C3, Cz, C4, T4, T5, P3, Pz, P4, T6, O1, O2) with an electrode cap using tin electrodes, referenced to A1. The cap was grounded by an electrode located midway between Fp1/Fp2 and Fz. Data were acquired using a Neuroscan Synamps 2 digital signal-processing system and Neuroscan 4.3.1 Acquire software, and the display and stimulus markers were controlled by a linked stimulus computer using Neurobehavioral Systems Inc. Presentation V 13.0 Build 01.23.09 software.

EOG was recorded using tin cup electrodes placed 2 cm above and below the left eye for vertical movements, and on the outer canthus of each eye for horizontal movements. Impedance was less than 5 kΩ for cap, EOG, and reference electrodes. Scalp and EOG potentials were amplified with a gain of 500 and digitized at a rate of 1000 Hz.

### Data extraction

The EEG data were EOG corrected using the RAAA EOG Correction Program (Croft and Barry, 2000). Single trial ERPs were re-referenced to digitally linked ears and extracted offline using the Neuroscan Edit software, low pass filtered (0.1–30 Hz, zero-phase shift, 24 dB/Octave), epoched -100–900 ms, and baseline corrected using the 100 ms pre-stimulus period. Trials containing omission (misses) or commission (false alarms) errors, or response times (RTs) longer than 800 ms, were excluded. Data were manually inspected for additional artifacts, and any contaminated trials were rejected, together with errors and RTs > 800 ms. An average of 2.12% trials (*SD* = 0.95) were excluded for young adults, and 2.02% trials (*SD* = 0.95) for older adults; these did not differ significantly *t*_(39)_ = 0.313, *p* = 0.756. For each subject, averages were computed for each of the four TTIs of interest. P3 peak amplitudes and latencies were obtained from these means for each subject and each of the TTIs, relative to the 100 ms pre-stimulus baseline; P3 amplitudes and latencies were also computed for nontarget means for each subject. Peak data were automatically selected and manually checked using a latency window of 280–420 ms for young adults, and 280–450 ms for older adults. These latency windows were decided based on the grand mean ERPs.

### Statistical analyses

The design was mixed, with a between-subjects factor of group (young vs. older) and within-subjects factor of TTI (1.5, 3.0, 6.0, and 9.0 s). Separate MANOVAs were carried out on P3 amplitudes and latencies with the above factors, with planned orthogonal contrasts to assess topography across the midline sites (Fz vs. Pz, and Cz vs. mean of Fz and Pz). Topographic distribution of P3 amplitudes and latencies can be examined efficiently by utilizing these orthogonal planned contrasts. Trends over TTI were assessed within subjects, using orthogonal polynomial contrasts with weighted linear and quadratic trends. The effect of stimulus type (target vs. nontarget) was examined by collapsing across TTI and using mixed MANOVAs between groups and across the midline sites separately for P3 amplitudes and latencies. Mixed MANOVA examined the effect of group and interval on RT; again with weighted linear and quadratic trend analyses. No Bonferroni-type α adjustment was required as *a priori* contrasts were used, and the number of contrasts did not exceed the degrees of freedom for effect (Tabachnick and Fidell, 1989). We also tested for any correlations between our three outcome measures (P3 amplitude and latency, and RT), with each subject and TTI as data points, separately for each group; Fisher *z* transformations were then calculated to compare groups. All *F*-tests reported have (1, 39) degrees of freedom.

It should also be noted that, as this paper details results for a number of dependent measures, the frequency of Type I errors increases. However, this increase in frequency of Type I errors cannot be controlled by adjusting α-levels, because the probability of Type I error remains the same (Howell, 1997).

## Results

To aid interpretation of results, trends analyzed across TTIs are denoted as “linear TTI” and “quadratic TTI.” The direction of difference between variables is indicated by “<” and “>”, and interactions between effects by “×”. Table 1 shows the relative change (%) in P3 amplitudes, latencies, and RTs *cf*. the 1.5 s TTI level, separately for young and older adults.

**Table 1:** **Percentage change in P3 amplitudes (μV) at Pz, latencies (ms) at Cz, and RTs (ms) to targets relative to the first TTI (1.5 s), separately for young and older adults**.

	**TTI**			
	**1.5 s**	**3.0 s**	**6.0 s**	**9.0 s**
**YOUNG**				
Amplitude	100%	123%	126%	131%
Latency	100%	101%	99%	102%
RT	100%	95%	94%	95%
**OLDER**				
Amplitude	100%	113%	113%	113%
Latency	100%	100%	97%	98%
RT	100%	97%	96%	94%

### Grand means

**Figure 1:**
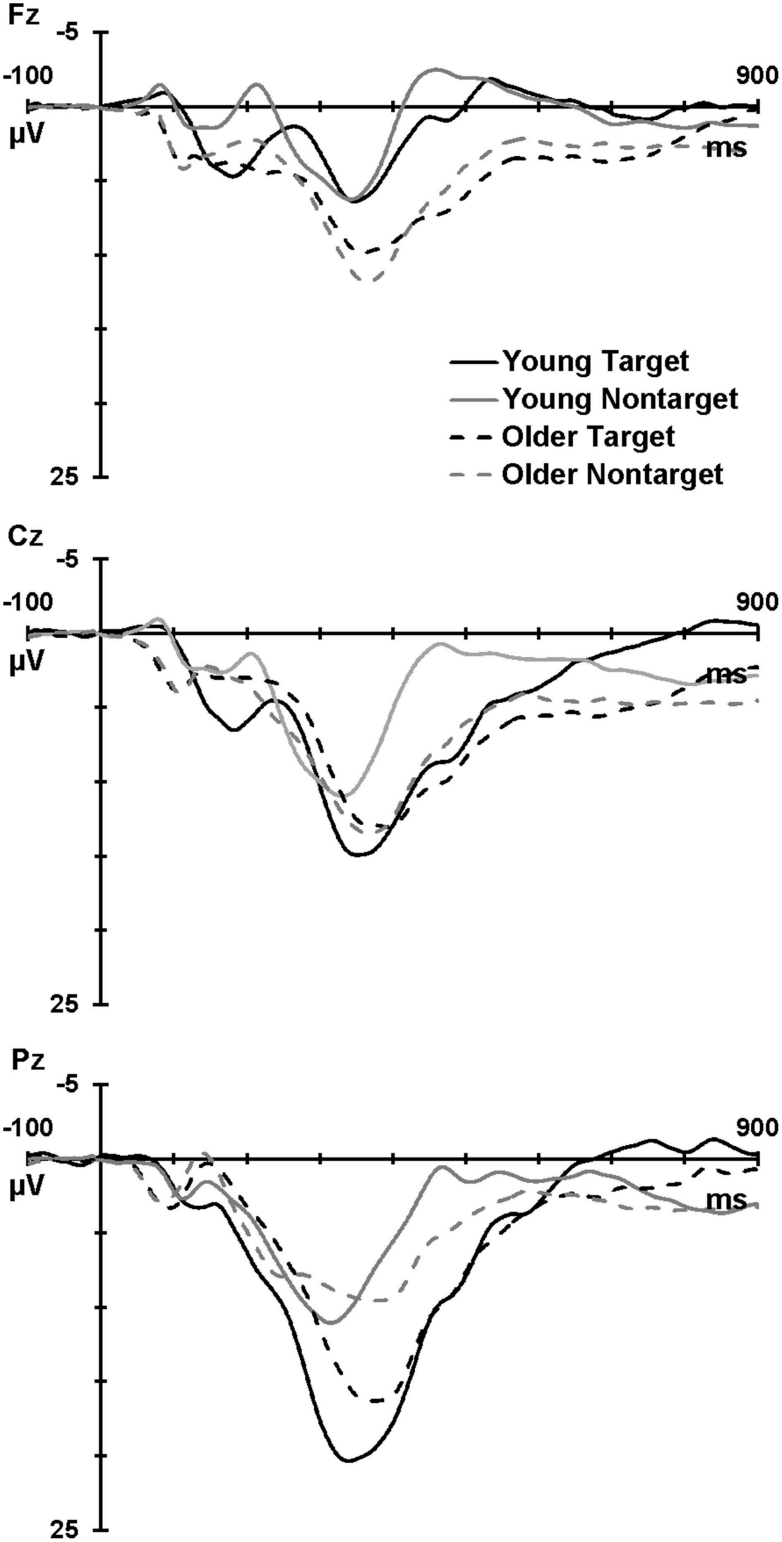
**Grand mean ERPs at Fz, Cz, and Pz for targets (black) and nontargets (gray) for young (solid line) and older (dashed line) adults**.

**Figure 2:**
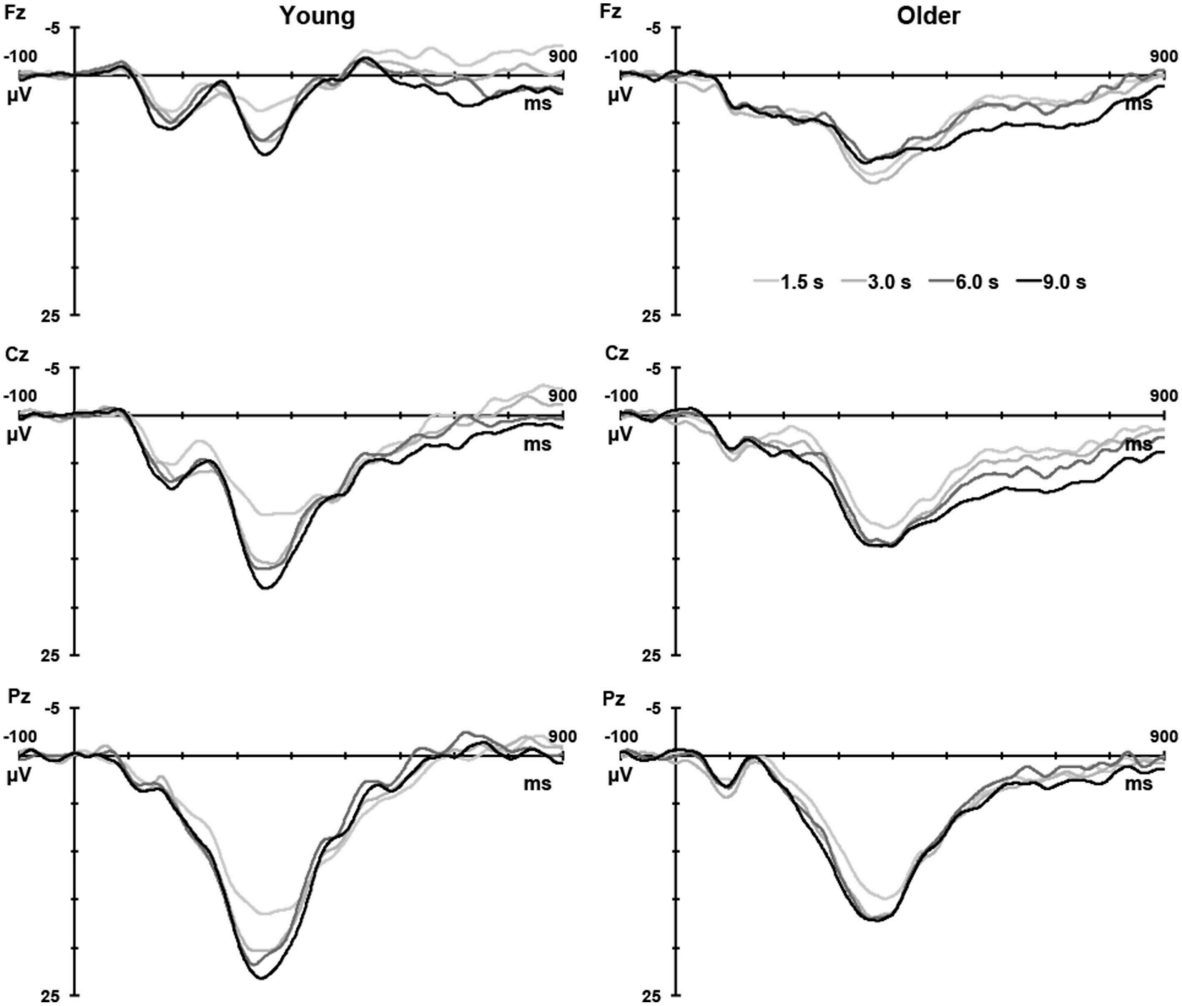
**Grand mean ERPs to targets for each analyzed TTI (1.5, 3.0, 6.0, and 9.0 s) at Fz, Cz, and Pz for young (left) and older (right) adults**.

**Figure 3:**
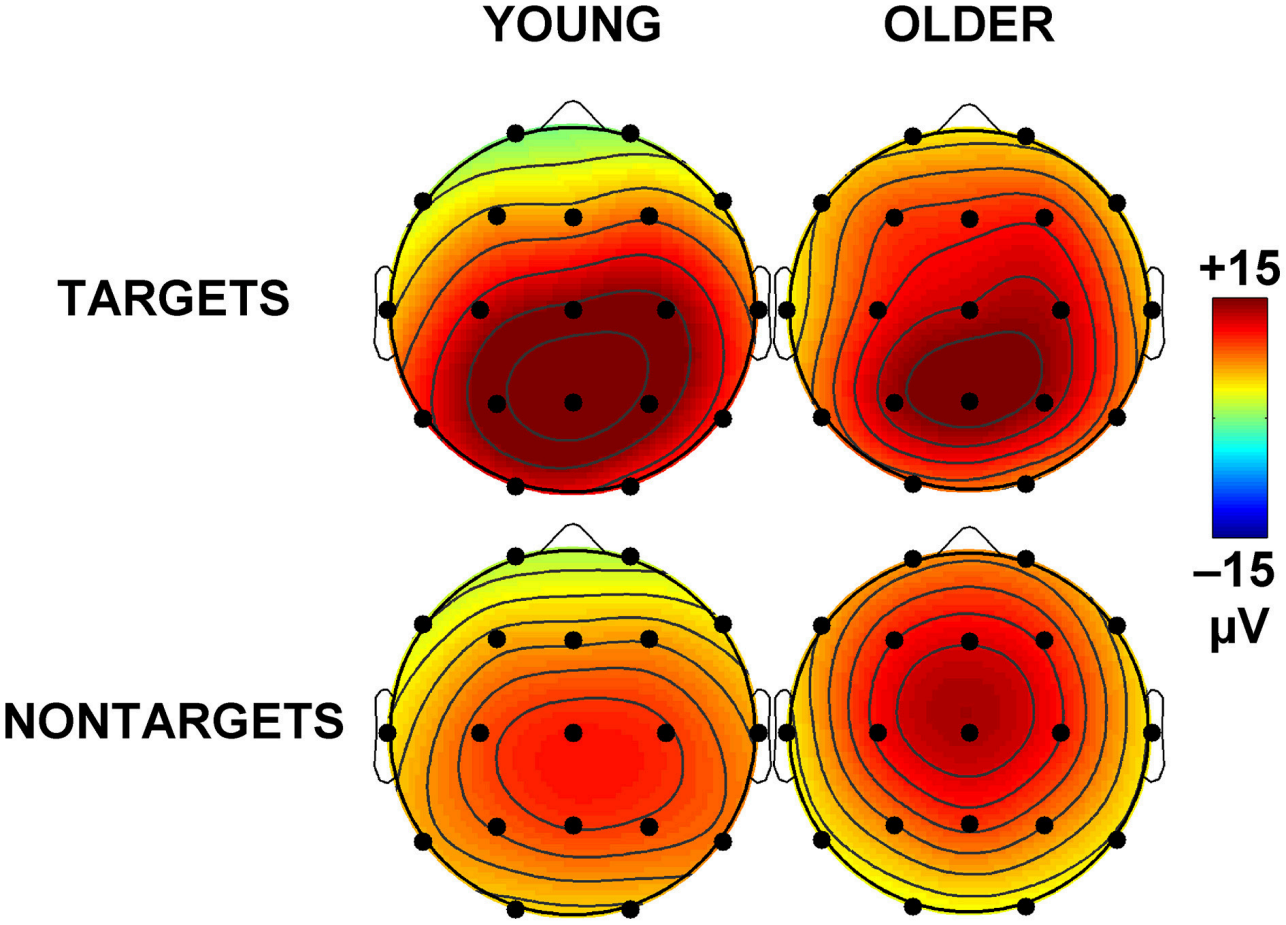
**Mean topographic headmaps for each stimulus category (top: targets; bottom: nontargets) and each group (left: young adults at 350 ms; right: older adults at 365 ms)**.

Figure 1 illustrates the grand mean ERPs for targets and nontargets from midline sites for young (solid line) and older (dashed line) adults. Grand mean ERPs for each of the four TTIs of major interest from midline sites are displayed in Figure 2 (young adults: left column; older adults: right column). Figure 3 shows grand mean headmaps for young (left) and older (right) adults, separately for targets (top) and nontargets (bottom); mean P3 amplitudes were taken at the midpoint of the latency window for young (350 ms) and older (365 ms) adults.

### P3 amplitude

Across group and TTI, P3 was larger at Pz than Fz (Pz > Fz: *F* = 230.08, *p* < 0.001, $\eta_{p}^{2}$ = 0.86), and at Cz compared the mean of Fz and Pz (Cz > mean Fz/Pz: *F* = 6.23, *p* = 0.017, $\eta_{p}^{2}$ = 0.14). These parietal and central enhancements were greater for targets than nontargets (Pz > Fz × target > nontarget: *F* = 124.29, *p* < 0.001, $\eta_{p}^{2}$ = 0.76; Cz > mean Fz/Pz × target > nontarget: *F* = 10.14, *p* = 0.003, $\eta_{p}^{2}$ = 0.21), and this contributed to a main effect of stimulus type (target > nontarget: *F* = 20.48, *p* < 0.001, $\eta_{p}^{2}$ = 0.34). At Pz, P3 was larger for young compared to older adults (Pz > Fz × young > older: *F* = 31.09, *p* < 0.001, $\eta_{p}^{2}$ = 0.44). Headmaps in Figure 3 also demonstrate that P3 was slightly more frontal for older than young adults, particularly for nontargets.

**Figure 4:**
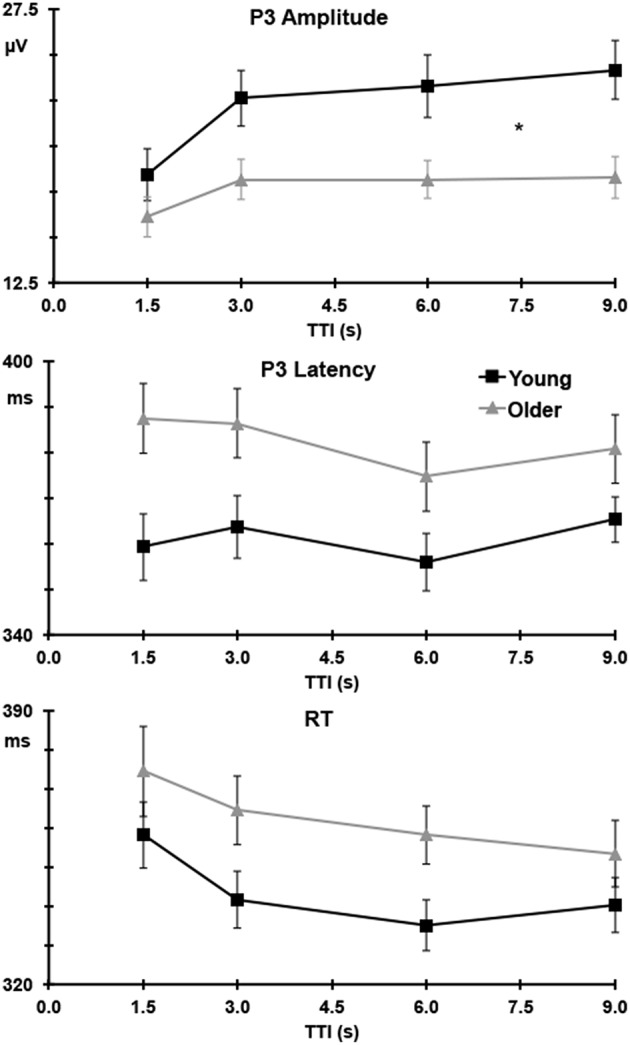
**P3 amplitude (upper) at Pz and latency (middle) at Cz to targets, and RT (lower) as a function of TTI with standard error bars; young adults are detailed in black and older adults in gray**. *For P3 amplitudes indicates a significant difference (*p* = 0.001) in the linear trend between young and older adults.

Figure 4 (upper) illustrates target P3 amplitudes as a function of TTI at Pz, separately for the young and older groups. Across groups and sites, P3 amplitudes increased as TTI increased (linear TTI: *F* = 17.57, *p* < 0.001, $\eta_{p}^{2}$ = 0.31), this plateaued at longer intervals (quadratic TTI: *F* = 5.44, *p* = 0.025, $\eta_{p}^{2}$ = 0.12). The linear increase was most apparent at Pz (linear TTI × Pz > Fz: *F* = 8.08, *p* = 0.007, $\eta_{p}^{2}$ = 0.17) and Cz (linear TTI × Cz > mean Fz/Pz: *F* = 21.36, *p* < 0.001, $\eta_{p}^{2}$ = 0.35). Figure 4 (upper) shows that the increase in P3 amplitudes with TTI increments was greater for young than older adults (linear TTI × young > older: *F* = 13.05, *p* = 0.001, $\eta_{p}^{2}$ = 0.25), this effect was largest at Pz (linear TTI × young > older × Pz > Fz: *F* = 4.20, *p* = 0.047, $\eta_{p}^{2}$ = 0.10). This can been seen as a relative change in Table 1, where P3 amplitude is stable for older adults after the second TTI level, but it continues to increase over intervals for young adults.

### P3 latency

P3 latencies were longest at Cz, compared to the mean of Fz and Pz (Cz > mean Fz/Pz: *F* = 18.88, *p* < 0.001, $\eta_{p}^{2}$ = 0.33). Latencies were longer for targets than nontargets (target > nontarget: *F* = 8.79, *p* = 0.005, $\eta_{p}^{2}$ = 0.18), and this delay was most apparent at Cz (target > nontarget × Cz > mean Fz/Pz: *F* = 13.97, *p* = 0.001, $\eta_{p}^{2}$ = 0.26). Figure 4 (middle) shows P3 latencies to targets over TTI for the young and older groups separately at Cz. Latencies were longer for the older compared to the young group (older > young: *F* = 7.07, *p* = 0.011, $\eta_{p}^{2}$ = 0.15). There was a trend toward a decrease in latency with increases in TTI at Cz (linear TTI × Cz > mean Fz/Pz: *F* = 3.36, *p* = 0.074, $\eta_{p}^{2}$ = 0.08), this did not differ between young and older adults; see Figure 4 (middle).

### RT

As shown in Figure 4 (lower panel), there was a trend toward slower RTs in the older compared to the young group (young < older: *F* = 2.87, *p* = 0.098, $\eta_{p}^{2}$ = 0.07). TTI significantly affected RT, with faster RTs to longer TTIs (linear TTI: *F* = 15.91, *p* < 0.001, $\eta_{p}^{2}$ = 0.29). This did not differ statistically with group, but Table 1 shows a more consistent relative decrease in RT for older compared to young adults.

### Correlations

Three separate bivariate correlations compared P3 amplitudes with latencies and RT, and P3 latencies with RT. There was no significant association for any of these 3 comparisons for either group, although there was a trend toward an inverse relationship between P3 amplitudes and RT for young adults, *r*_(74)_ = −0.159, *p* = 0.086, one-tailed, and between P3 latencies and RT, *r*_(74)_ = −0.153, *p* = 0.094. Fisher transformations revealed no significant differences for any of these comparisons for young compared to older adults.

## Discussion

This work compared TTI effects on the P3 in older and young adults. Results were in line with expectations that young adults would show a greater TTI effect than older adults. These findings suggest that older adults have weaker electrophysiological memory-updating processes than young adults, as interpreted by the template-update model.

### TTI effects and memory-updating

P3 amplitudes were augmented by longer TTIs in both young and older adults; a finding in line with all previous TTI studies (Gonsalvez et al., 1999, 2007; Gonsalvez and Polich, 2002; Croft et al., 2003; Steiner et al., 2013a,b; Steiner et al., 2014a,b). As hypothesized, the linear increase in P3 amplitude with longer TTIs differed with age, with young adults showing a steeper increase than older adults. The relative change in P3 amplitudes (*cf*. the first TTI level) clearly demonstrates this with amplitudes increasing to 131% of their initial value by the longest TTI for young adults, but increasing to only 113% for older adults by the second TTI level, before stabilizing at that magnitude. These findings are consistent with the notion that the P3 component captures updating processes following degradation of target templates in working memory, as the template-update model suggests (Gonsalvez et al., 2007), and that this process is compromised in older adults.

From the template-update perspective, the practice of averaging P3 amplitudes across TTIs is flawed because P3 represents an update process that can be meaningfully captured only by a function across time (as shown in Figures 2, 4). Because updating processes can be affected by several factors including encoding strengths (e.g., salient stimuli, stimuli of higher intensities, and stimuli that activate networks with rich associations) and differential slopes of template decay, P3 values at longer TTIs (especially if the value demonstrates the peak reaching asymptote) are of significance, because the value represents the completion of updating and a potential measure of encoding strength. On the other hand P3 values during intermediate TTIs could represent the effect of two or more processes (stimulus encoding strength and decay). Within this context, smaller amplitudes at longer TTIs (e.g., 9 s) would suggest inadequate encoding (weaker encoding, fewer or less effective activation of associative networks) of target stimuli in the older group. These results are consistent with literature suggesting that the ability to maintain templates required for stimulus categorization decreases with age (Fabiani and Friedman, 1995).

Both decreased P3 amplitudes and increased P3 latencies are reliable predictors of decline of cognitive processing in aging and dementia. The current study clarifies these results by demonstrating that the indices that best differentiate the two groups are P3 amplitudes derived from longer TTIs (template-encoding differences) and P3 latencies at shorter TTIs (delayed stimulus categorization or recovery functions for template formation). In addition, RTs were faster with increases in TTI, and there was a trend toward the same pattern of results for P3 latency across both age groups; a finding consistent with previous work (Gonsalvez and Polich, 2002; Gonsalvez et al., 2007; Steiner et al., 2013b). There were no significant correlations between P3 amplitudes and latencies, between P3 amplitudes and RTs, or between P3 latencies and RTs for both groups, suggesting that P3 amplitude over TTI is an independent index of memory-updating processes.

Older adults showed an overall reduction in processing power and longer processing times compared to young adults. This was evidenced by reduced P3 amplitudes, longer P3 latencies, and a trend toward slower RTs in older vs. young adults. These are well-established findings (Polich, 1997; West et al., 2010; Barry et al., 2016) that are also accompanied by an anterior shift in P3 topography (evident in Figure 1 at Fz and in Figure 3) due to a reduction in processing in the parietal cortex and an increased reliance on hippocampus and prefrontal structures including dorsolateral prefrontal cortex (O'Connell et al., 2012). This compensatory processing also takes more time, resulting in slower RTs (Falkenstein et al., 2006). Our pattern of results is consistent with the Scaffolding Theory of Cognitive Ageing, where neuroplasticity alleviates the pathophysiological cognitive decline associated with aging (Goh and Park, 2009). Here, the brain responds to age-related changes in anatomy and physiology (e.g., reductions in cortical thickness, dopaminergic activity, white matter integrity) by recruiting additional regions of activation and increasing its activity in order to meet the demands of information processing. This is evidenced in our study as both groups responded with similar task accuracy (2.12 vs. 2.02% errors), but the older group showed a more diffuse distribution of P3 amplitudes (see Figure 3; positivity indicated in red is distributed more broadly across the scalp).

### Limitations

Because the primary focus was to study the effects of TTI as a function of age, TTI manipulations using a single stimulus were considered. Such a paradigm was trialed initially but abandoned because several subjects, especially older adults, tended to become drowsy when targets were separated by long intervals of silence (e.g., 9 s). The task structure utilized in the current study could be optimized in future work. Paradigms using longer TTIs (e.g., 15 s; Steiner et al., 2013a) are more sensitive for capturing memory-updating differences and would be more suitable as potential predictors of aging and dementia. In addition, this two-stimulus task with a fixed ISI meant that TTI was confounded with stimulus-sequence. Future work could disentangle these effects by utilizing a three-stimulus task, or randomly varying the ISI around a 1.5 s mean. Further research examining the relationship between cognitive measures and the various subcomponents of the P3 is also warranted. It should also be noted that we did not obtain the level of education of participants, however all participants were well functioning, understood the task instructions (as indicated by their high performance), and could read and write in order to provide informed consent.

## Conclusion

This study examined TTI effects on the P3 in young and older adults. We found that increases in TTI enhanced P3 amplitude in a linear fashion, and this effect was weaker for older adults. There was also an overall decrease in P3 amplitudes, a slowing of latencies and RTs, and an anterior shift in topography in older adults. Findings indicate that memory-updating processes are compromised in older adults, with a poorer ability to encode, maintain, and update stimulus templates in working memory. Further work is needed to ascertain whether people living with dementia or Mild Cognitive Impairment (MCI) show a different pattern of results.

## Author contributions

GS analyzed the data and drafted the manuscript. CG designed the study, liaised with the aged care facility, supervised the project, and provided critical feedback on the drafted manuscript. FD collected the data. RB supervised the project and provided critical feedback on the drafted manuscript.

## Funding

Thank you to the Illawarra Health and Medical Research Institute (IHMRI) for funding this research.

### Conflict of interest statement

The authors declare that the research was conducted in the absence of any commercial or financial relationships that could be construed as a potential conflict of interest.

## Abbreviations

EEG: Electroencephalogram
EOG: Electrooculogram
ERPs: Event-related potentials
MCI: Mild Cognitive Impairment
RT: Reaction time
TTI: Target-to-target interval.
